# The Defensive Behaviors and Milk Production of Pastured Dairy Cattle in Response to Stable Flies, Horn Flies, and Face Flies

**DOI:** 10.3390/ani13243847

**Published:** 2023-12-14

**Authors:** Anna C. Hansen, Roger D. Moon, Marcia I. Endres, Glenda M. Pereira, Bradley J. Heins

**Affiliations:** 1 Department of Entomology, University of Minnesota, Saint Paul, MN 55108, USA; hans4863@umn.edu (A.C.H.); rdmoon@umn.edu (R.D.M.); 2 Department of Animal Science, University of Minnesota, Saint Paul, MN 55108, USA; miendres@umn.edu (M.I.E.); glenda.pereira@maine.edu (G.M.P.)

**Keywords:** stable fly, horn fly, face fly, behavior, pasture, milk production

## Abstract

**Simple Summary:**

Fly control has always been a hot topic for dairy farmers, because there are not a lot of viable options to alleviate fly pressure. Dairy cows on pasture are commonly disturbed by many fly species, and dairy cow behavior may be affected by different fly species. In an experimental research trial, there was a strong relationship between the number of flies and the number of defensive behaviors of cows of different lactations. Milk yield was not affected by low fly numbers, indicating that greater than 40 horn flies per cow are needed to potentially lower the milk production of grazing dairy cows. To improve profitability, farmers need to properly identify key pasture flies, understand their biology and habitat, monitor their populations, and then reduce the fly population through mechanical or biological management techniques.

**Abstract:**

Thirty-four crossbred dairy cows were observed on pasture six times per week from June to August 2014 at the University of Minnesota West Central Research and Outreach Center grazing dairy in Morris, MN, for defensive behaviors in response to three species of muscid flies. Counts of stable flies (*Stomoxys calcitrans* (L.)), horn flies (*Haematobia irritans* (L.)), and face flies (*Musca autumnalis* DeGeer) were recorded before and after pasture observation. Individual cows were monitored for 5 min intervals to observe the frequencies of five different defensive behaviors: front and back leg stomps, head tosses, skin twitches, and tail swishes. Fly numbers averaged 5 stable flies per leg, 37 horn flies per side, and 1 face fly per face during the study. The fly counts and behavior frequencies increased with ambient temperature. The results showed a very strong relationship between the numbers of flies and numbers of defensive behaviors, though correlations between specific flies and behaviors were low. Younger cows had fewer stable flies and horn flies than older cows. The thresholds of flies to lower production for pastured organic dairy cows may be greater than 5 for stable flies, 37 for horn flies, and 1 for face flies.

## 1. Introduction

Cattle have a myriad of associated parasites, including several species of flies. Biting flies on pastured dairy cows may introduce challenges that may decrease the animal welfare of cows [1,2]. Defensive behaviors in response to flies not only interrupt grazing, but can increase the energy costs of grazing [3,4,5]. Stress from flies can lead to serious production losses in feed efficiency, weight gain, and milk production. The most important pest flies in dairy are muscid flies and include stable flies, horn flies, and face flies. 

Flies are present wherever there are cattle and have long been a problem for livestock producers. Nuisance flies are typically most active from May to October in northern regions and are active year-round in warmer climates. These flies feed on cattle, whether housed indoors or on pasture. 

Cows are irritated by fly feeding behavior and can become very stressed under excessive fly populations. Cattle waste energy removing flies, which leads to reduced grazing times. Cows also become restless and spend less time lying down when under heavy fly pressure, and cows might have a decreased feed intake due to the disturbance of defensive behaviors. Prolonged exposure to fly irritation may lead to a decrease in milk production. Although flies cannot realistically be eliminated from a farm, producers benefit from fly management, with more comfortable animals and people. Proper management can keep fly populations in check to minimize their negative effects [6,7].

Weather is an important factor in the activity and feeding behaviors of flies, although results describing how weather affects this activity are variable. Temperature is consistently an important variable for fly activity. The feeding activity of stable flies ceases at temperatures below 15 °C [8]. Smith and Hansens [9] fed stable flies at varying combinations of temperature and humidity in the lab and found the highest percentage of stable flies feeding at 32 °C with a relative humidity below 43%. Furthermore, the lowest percentage of flies was found feeding at 23 °C with a relative humidity above 75%. Berry and Campbell [10] noted that, while stable fly feeding behavior was influenced by varying weather effects, feeding was partially dictated by time of day, regardless of weather.

Stable flies (*Stomoxys calcitrans* (L.)) are blood-feeding flies typically found on the legs of cattle. These flies were long considered to be pests of confined cattle, but the introduction of round bale hay feeders has resulted in increased populations on pastured cattle [11]. Horn flies (*Haematobia irritans* (L.)) are another blood-feeding species, which are most often found on the backs, sides, or bellies of cattle. Horn flies feed multiple times per day and spend almost all of their adult stage on their host. Heavily infested animals can host several thousand horn flies at any given moment. Face flies (*Musca autumnalis* DeGeer) are nonbiting flies that feed on liquid secretions, typically around the eyes and muzzle of cows. These flies cause irritation and can vector eye-inhabiting parasites and pathogens [7]. 

Fly presence encourages cows to move more frequently to newer areas [12] and alter their grazing bouts. Defensive behaviors not only interrupt grazing, but can increase the energy costs of grazing [3]. Cows annoyed by flies exhaust energy once directed toward production in an attempt to dislodge the flies [13]. The intensity of attacks varies with time of day and weather conditions. Flies are particularly active when winds are low and temperatures are high [14]. Under intense attack, cows often abandon grazing and bunch close together. Bunching is a herd response to fly activity where cows attempt to limit the surface area exposed to attack. Oftentimes, cows will gather in a tight circle with their heads in the center. 

Quantifying defensive behaviors in response to flies is useful for producers planning the best management strategies to increase production. However, most studies focus on a single pest fly species. Oftentimes, cattle are infested with multiple species, making it difficult to attribute effects to a specific fly [15]. Nuisance flies and the defensive behaviors exhibited by cows in response to these flies are easily observed interactions. Previous studies have focused on how cow behavior is affected by a single species of fly. Dougherty et al. [3,4,5,16,17] released starved laboratory-raised stable flies on grazing beef cattle to observe the behavioral responses to fly feeding behavior. Mullens et al. [15] documented stable flies on dairy cows in a feedlot throughout the fly season to assess the relationships between stable flies, defensive behaviors, and milk production. Also examined were the effects of face flies on cattle behavior, individually and as a herd [18,19], and these studies showed that pest flies can increase the frequencies of these defensive behaviors, with many concluding that production was negatively affected by fly infestations. However, results showing the extent to which production is reduced are variable. For example, Bruce and Decker [20] found that stable flies suppressed milk production well past the end of the fly season, while Mullens et al. [15] was unable to detect effects on milk production. 

Mullens et al. [15] monitored four groups of 25 cows twice a day, five times per week, over 12 weeks to study the behavioral responses to stable flies on cows housed outdoors in a dirt lot. Stable flies were counted, and then responses (head throws, leg stamps, skin twitches, and tail flicks) were recorded for two minutes. After the observation period, the stable flies were recounted. The stable fly numbers varied between individual cows and the cows differed in their response to attack [15]. Cows subjected to harassment throughout the fly season showed decreases in leg stamping and head throws, indicating habituation to bites [15]. Warnes and Finlayson [21] found that cows earnestly exhibiting defensive behaviors were attacked by fewer flies than calmer animals. Front leg stamps are a good indicator of stable fly presence, though tail flicks may be easier for producers to observe and decide on a treatment protocol as part of an integrated pest management strategy [15]. 

Cows infested with horn flies differ in grazing behavior from un-infested cows. Cows not disturbed by horn flies were spread more widely in pasture. Harvey and Launchbaugh [22] noted that steers infested with 300 or more horn flies tended to walk more and differed in grazing/rumination behavior compared to steers without horn flies. They stocked two pastures with nine yearling Hereford steers, one herd controlled for horn flies and one herd without control. Over nine days, the activity from all steers was recorded from morning rise to bedding down at night. Tail flicks were recorded for 2 min at 15 min intervals. Behaviors such as leg stamps or head throws were recorded intermittently. Infested steers had significantly more tail flicks than steers treated for horn flies. Other physiological responses to horn fly infestations include increased heart rate, respiration, temperature, and cortisol levels [23]. Harvey and Launchbaugh [22] concluded that infested steers likely have an increased energy requirement, which would need to be offset by increased feed intake or feed efficiency. 

The feeding habits of face flies annoy cattle, as evidenced by observed defensive behaviors. Irritation from face flies alters grazing behavior, reducing energy intake [4]. Ear flaps in particular are a good indicator of face fly presence. According to Schmidtmann [18], ear flaps are adaptive behaviors that interrupt face fly feeding, in that fly numbers are greater before flaps than after. In their study, face flies were counted every 20 s, along with the numbers of ear flaps during that interval. Behavior was recorded on both the right and left side by two observers positioned from 3 to 5 m away from cattle. 

When face fly numbers are high, cattle may be seen bunching, defined by Schmidtmann and Valla [19] as bouts exceeding 15 min where at least eight heifers are positioned in a circle with their heads pointed medially. To better understand the relationship between face fly numbers and herd density, Schmidtmann and Berkebile [24] observed seven herds of 14–16 cows 3 times per day over 10 consecutive day periods. During that time, face flies were counted and proximity to another cow was recorded on a scale from 0 (0–0.5 m distance) to 3 (>3 m distance). Cows protecting their faces tended to have lower numbers of face flies [19,24].

With many fly species associated with cattle, associating a species with a specific behavior can be difficult when observing free-roaming cattle in pasture. Behaviors are easier to observe from a distance than flies, and so knowing the relationship between defensive behaviors and fly numbers can be a useful tool for producers. Rather than counting flies, a producer could potentially observe cows for a brief period to estimate if fly management is necessary to maintain production. Therefore, the objective of this study was to determine the muscid fly populations on two groups of pastured cattle. Furthermore, fly counts were compared with observed behavior frequencies to understand how cows respond when attacked by multiple fly species simultaneously. Finally, fly counts and defensive behaviors were compared with electronically recorded milk weights to assess the associations among fly counts, frequencies of behavior, and milk production.

## 2. Materials and Methods

### 2.1. Research Location

This study was conducted with the certified organic dairy herd at the University of Minnesota’s West Central Research and Outreach Center (WCROC) in Morris during summer 2014. The site housed independently managed herds of organic and conventional crossbred and Holstein cattle. Pastures suitable for grazing surrounded the milking parlor. Two independently managed groups of certified organic crossbred dairy cows were studied and each group consisted of 17 cows, balanced by breed, parity, and milk production. 

The cows were turned out to pasture on May 28 and remained on pasture throughout the summer, except when being milked twice per day. The groups grazed primarily on cool season grasses, including smooth bromegrass (*Bromus inermis*), red clover (*Trifolium pretense*), white clover (*Trifolium repens*), meadow fescue (*Festuca pratensis*), perennial ryegrass (*Lolium perenne*), and alfalfa (*Medicago sativa*). Observation days occurred three times each week, with the cows being observed twice within that day, except during extreme heat conditions. The lack of data collection may have affected the results, but the safety of the observers was taken into account based on high heat conditions. The study began on June 5 and concluded on August 15. The cows were milked in a swing-9 para-bone milking parlor at 06:00 and 17:00. Milk weights were electronically recorded for each cow at every milking. 

Weather records were obtained from the WCROC weather station to assess the associations of temperature, wind speed, relative humidity, and/or precipitation during the observation periods with fly counts and behaviors. Temperatures were recorded during all observation periods, as well as the observation day’s minimum, maximum, and mean temperature. 

### 2.2. Fly Counts and Defensive Behaviors

Both groups of cows were observed during summer to measure the fly abundance and concurrent frequencies of defensive behaviors. Cows were observed between 9:00 and 11:00 a.m., and again between 1:00 and 3:00 p.m. Two observers were used during each period so both groups could be observed simultaneously. Individual cows were identified with numbered ear tags. The cows were observed from a distance from 1 to 2 m to allow for accurate fly counts without disturbing the cows’ natural behaviors. 

An observer would approach an individual focal cow as much as possible, then count and record the number of muscid flies present on the cow. Stable flies were counted separately on the front and back legs. Leg counts were defined as the number of flies visible from brisket to hoof when viewed from a single angle where both legs were visible [25,26]. Horn flies were counted along one side, from the back and withers to the belly. Face flies were counted on faces, viewed head on. 

After counting the flies, the focal cow was observed for five minutes to tally its defensive behaviors. A stopwatch was used to keep track of the time, and behaviors were tally marked on a data sheet. The behaviors recorded were head throws, front leg stamps, back leg stamps, skin twitches, and tail flicks, using the definitions found in Mullens et al. [15] and Dougherty et al. [4]: Head throw: the nose crosses the transverse plane at the front of the chest on the observer’s side. Front or back leg stamp: either the front or back leg lifts enough to clear the ground while the animal is not walking. Skin twitch: a skin ripple about 2 s or more. Tail flick: the tail tip moves forward enough to cross an imaginary plane across the rear of the animal.

After five minutes, the flies were counted again, and then the pre- and post-observation counts were averaged to characterize abundance during the observation period. These processes were repeated until all the cows were observed. The observations were compared with the next day’s recorded milk production, presuming that any stress effects would be observed on the following day. Prior to the initiation of the study, fly counts were agreed upon in the pasture by observers. These methods for counting the flies on the cows were based on previous research studies. Differences between observations were negligible at the initiation of the study. Over 1700 observations were included in the data analysis.

### 2.3. Variation in Fly Counts and Behavior Frequencies

The hypothesis was that more flies would be observed in the afternoon than in the morning, and that counts would increase with temperature and humidity. For analysis, a linear mixed effects model was used with observer, date, parity (cows of different lactations), fly species, time of day (morning vs. afternoon), and time (start vs. end count) as fixed effects. Temperature, relative humidity, wind speed, and precipitation were included as covariates in the initial model, and groups and cows within groups were included as random effects. 

Analyses using the same methods were also performed for individual fly species. In the case of stable flies, location (forelegs versus hindlegs) was added as a fixed effect to determine if more stable flies were found on the front or back legs. 

To test the hypothesis that behavior frequencies would increase with fly counts, the counts of all five behaviors combined with the counts of all three fly species combined were compared. After detecting a significant interaction between time and observer, fly counts were adjusted to account for observer differences when analyzing behavior frequencies. For analysis, a linear mixed effects model was used with observer, date, parity, and fly species as fixed effects. Temperature, relative humidity, wind speed, and precipitation were also included as fixed covariates, with groups and cows within groups as random effects. A polynomial term for fly count was also included in the model to test for curvilinearity or saturation. 

Observation days were grouped into three time periods at the beginning, middle, and end of the study to test the hypothesis that cows became habituated to fly activity. The previously described analysis was repeated, replacing date with time period. The hypothesis was that if cows were becoming habituated to flies, there would be more defensive behaviors in the first time period at the beginning of the study and fewer behaviors in the third time period. 

These analyses were also performed for individual fly species to determine if any given species was a cause of greater irritation. In the case of stable flies, the counts from the front and back legs were added together to remove the effect of location, our rationalization being that stable fly presence causes irritation regardless of location. Analyses were then repeated for individual behaviors, beginning with a full linear mixed effects model and simplification when possible. 

To test the hypothesis that milk production decreased with increasing fly counts and behavior frequencies, electronically recorded milk weights from the day after each observation day were compared with the fly counts and behaviors observed the day before. The milk weights from the morning and afternoon milking were added together to calculate the daily milk production. Associations were determined between parity, days in milk (DIM), fly counts, fly species, behaviors, and temperature on the next day’s recorded milk production, presuming that stress effects would be observed the following day. 

### 2.4. Statistical Analysis

The experimental unit was cows for all analyses. An analysis of covariance examined the variation in the numbers of counted flies, observed behavior frequencies, and milk production. For each response variable, summary statistics were examined, followed by an examination of variation in relation to fixed and random effects. To start, a full repeated measures model was used with the fixed effects of observer, date, fly species, parity, and the interactions of these variables, and the random effects of groups and cows within groups. Random effects that accounted for less than 15% of the total variation were removed to conduct a simple analysis of covariance with just fixed effects. Appropriate 3- or 4-way interaction terms (interactions that included observer, date, fly species, and parity) were used in denominators to conduct conservative F-tests. Insignificant fixed effects and interaction terms were removed (*p* > 0.35) to create a minimally sufficient model. Interaction plots were constructed to evaluate the nature and magnitude of interactions. If an interaction was small in magnitude, then it was removed to further simplify the model. 

Added variable plots were used to graphically examine the relationships between the response variables of interest and continuous predictors after adjusting for other factors in the chosen model. Diagnostic plots were created to check the analytical assumptions of the final model. Log transformations of the fly counts and behavior frequencies were used as needed to satisfy the analytical assumptions of equal variance and normal distribution in errors. A graphical inspection of the residual plots confirmed these assumptions. All the statistical analyses were conducted using R 3.0.2, with packages “lme4”, “nlme”, and “car” (R Foundation for Statistical Computing, Vienna, Austria). The sample size for this study was determined with SAS software 9.4. The estimated sample sizes needed to achieve a power of > 0.80 for the behavior measurements were 15 cows per group. Over 1700 fly counts were included in the statistical analysis.

## 3. Results

The study occurred between 5 June and 15 August 2014, and included 27 observation days, with over 3000 fly counts and 1500 behavior observations. The first observation day on June 5 included observer training for primary observers and alternates, and therefore was removed from the analyses. Throughout the first week of July, both groups were transferred to a different pasture due to limited forage. Observations did not take place during that time and resumed on July 10 when the cows were moved back to their original pastures. One afternoon observation period in July was postponed due to severe lightning. A subset of data including observation days with only primary observers was analyzed after detecting a significant difference between the observers.

**Figure 1:**
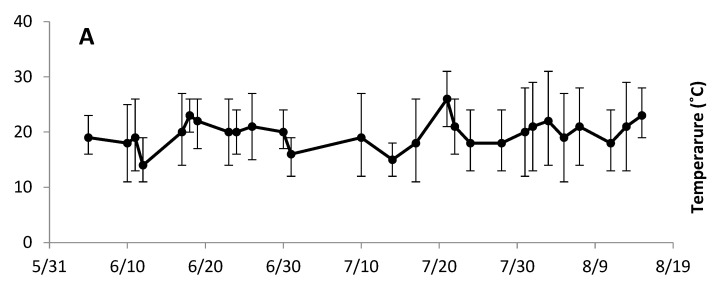

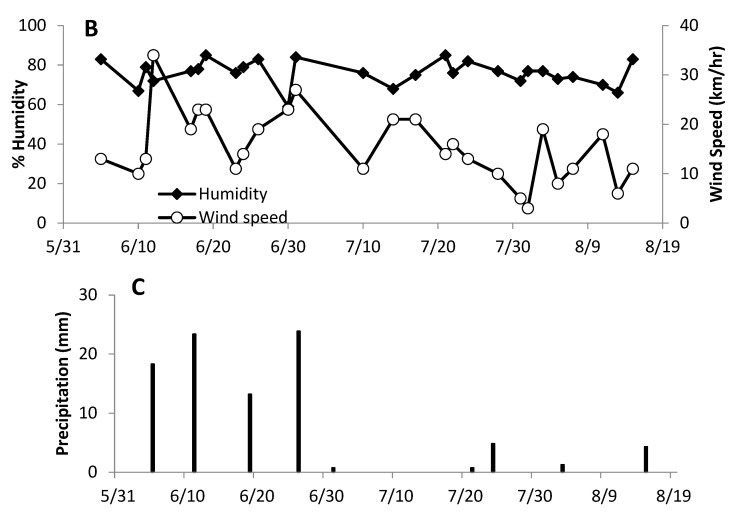
Weather conditions in Morris, MN, during summer 2014 on 27 group observation days. (**A**) Mean temperature with daily minimum and maximum temperature shown in error bars. (**B**) Daily average relative humidity and wind speed. (**C**) Daily total precipitation.

Temperatures ranged from 11 °C to 29 °C, with a summer average of 19.7 °C (Figure 1A). The mean temperature was 19.7 °C during the morning observations and 23 °C during the afternoon observations. Humidity ranged between 59 and 85% and average daily wind speed ranged from 3 to 34 km/h (Figure 1B). Daily precipitation ranged from 0 to 5 mm, with the exception of 4 days, where precipitation ranged from 13 to 24 mm in a day (Figure 1C). Weather trends were overall consistent with the ten-year averages at the research site. 

### 3.1. Variation in Fly Counts

**Figure 2:**
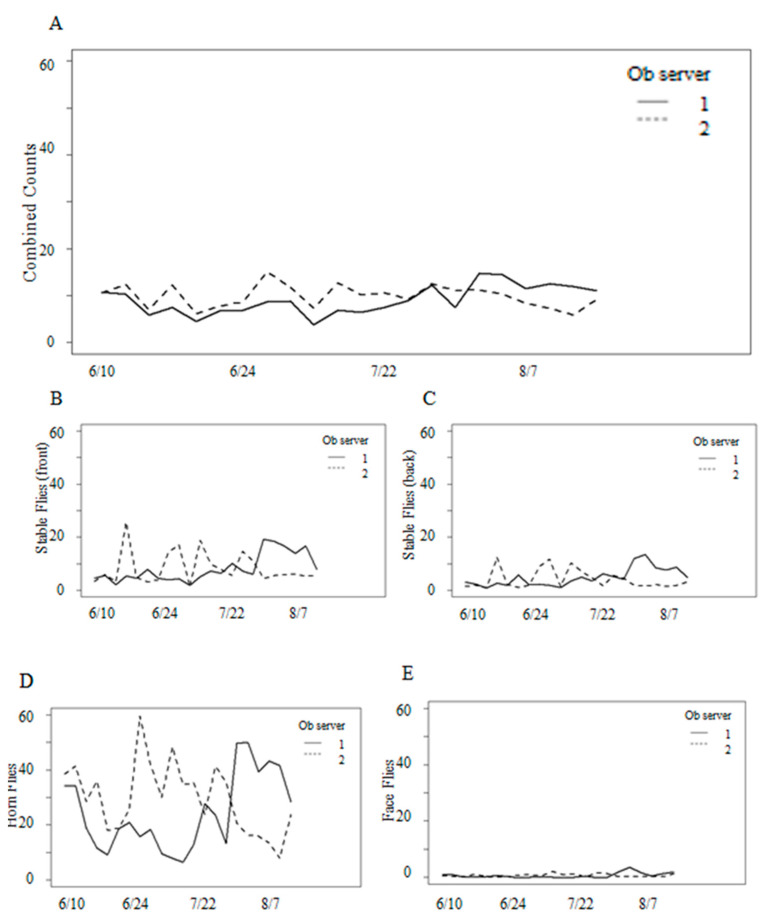
Daily average fly counts by date and for observer 1 and 2: (**A**) combined counts of all three species, (**B**) stable flies on front legs, and (**C**) back legs, (**D**) horn flies per side, and (**E**) face flies per face.

Formal analyses of the total combined fly counts indicated that the random effects of groups and cows within groups accounted for less than 15% of the total variation. Consequently, these effects were removed, and the analysis proceeded with fixed effects only. There was a significant interaction between observer and date, which indicated that the observers counted different numbers of flies as the study progressed (Figure 2A). Interaction plots showed that Observer 1 typically recorded more fly numbers than Observer 2, except for in the last six observation periods (Figure 2A). The differences in the daily means between observers were usually between 0 and 6 flies. 

**Figure 3:**
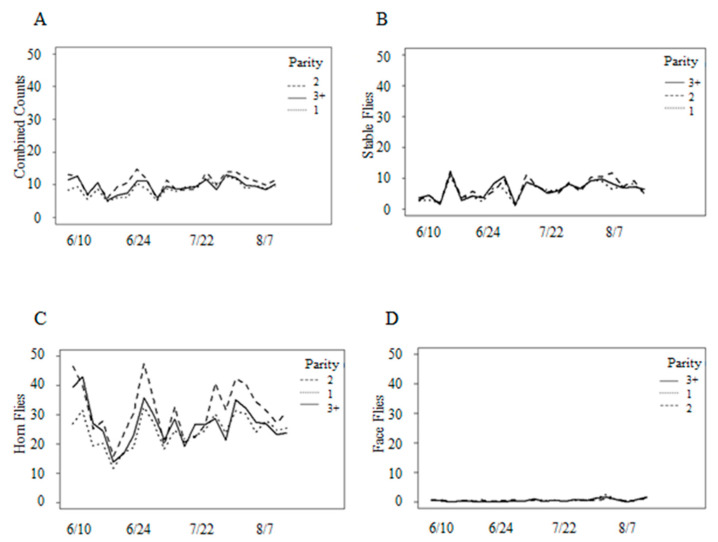
(**A**) Combined counts of three muscid fly species by cow parity (1st, 2nd, and 3+ lactations), (**B**) stable fly, (**C**) horn fly, and (**D**) face fly.

Fly numbers were lower on July 14 (Figure 3A). Temperatures were cooler at 12 °C in the morning and 16 °C in the afternoon, with average winds of 21 km/h and 68% humidity. Similarly, stable fly numbers were noticeably low on June 12 (Figure 2B,C), with the temperature at 10.5 °C and 16 °C in the morning and afternoon, respectively, and winds averaging 34 km/h and 72% humidity.

The total combined fly numbers covaried significantly with temperature, precipitation, and wind speed, but were independent of relative humidity (*p* = 0.24). Humidity was subsequently removed from the model. Fly counts were slightly higher in the afternoons, though this difference was not statistically significant (*p* = 0.33), and so fly counts were calculated as daily averages for use in further analyses. There were no significant differences in fly counts before or after the observation period (*p* = 0.91). Consequently, this effect of time was removed from the model and counts adjusted for observers were used in subsequent analyses.

Combined fly counts were variable with parity, despite statistically significant differences in the fly numbers for cows of different parities (Figure 3A). Between mid to late June and early to mid-August, cows in their second lactation hosted, on average, an additional one to five flies than other cows. In terms of fly species, the differences were approximately 5–15 additional horn flies for cows in their second lactation throughout most of the study (Figure 3C), and approximately 1–4 additional stable flies for cows in their second lactation during mid-August (Figure 3B). Significant interactions (*p* < 0.001) between species and parity, as well as species and date, indicated a need to analyze the variation in numbers for individual fly species.

**Figure 4:**
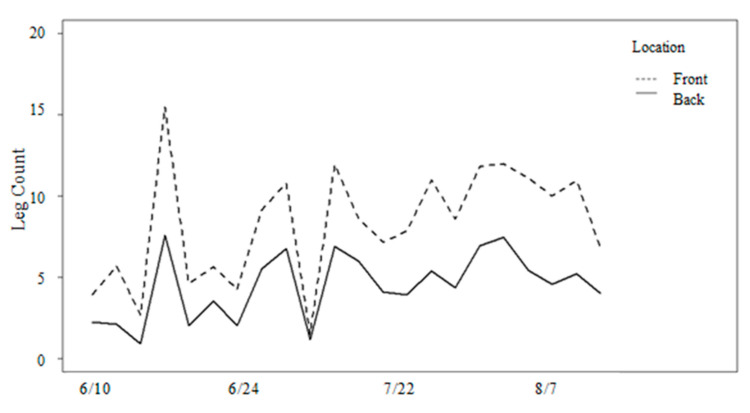
Mean number of stable flies counted on front and back legs in summer.

Stable fly activity began in the first full week of May, approximately one month before the study began. Stable flies averaged three flies per leg and gradually increased in numbers over the summer (Figure 2A,B). Analyses of stable fly numbers indicated that the daily average on the front legs ranged from 1 to 15 flies and from 1 to 7 flies on the back legs throughout the study (Figure 4). A significant difference of approximately twice as many stable flies on the front legs than the back legs throughout the study was observed (Table 1). Fly numbers differed significantly with parity (Table 1), with cows in their first lactation hosting fewer flies throughout most of the study (Figure 3B). Fly numbers significantly increased with temperature and precipitation but decreased with humidity (Table 1). Wind was not significant to stable flies (*p* = 0.59). An examination of added variable plots indicated that these relationships were weak. There was no significant difference in counts from the morning and afternoon (*p* = 0.43), nor in start versus end count (*p* = 0.47), so those effects were removed.

The daily average numbers of horn flies ranged from 0 to 150 per side. Throughout the duration of the study, cows hosted a mean of approximately 35 horn flies (Figure 2C), with an observed increase in fly numbers as temperature increased. In the range of temperatures recorded during this study, we observed an increase of 1.2 horn flies with every 2° increase in temperature. Parity was a statistically significant factor for horn fly counts (*p* < 0.001), with fewer flies on first lactation cows for most of the study (Figure 3C). Counts decreased with increasing wind speeds and precipitation but increased with humidity (Table 2). However, an examination of added variable plots indicated that these relationships were weak. More horn flies were observed in the afternoon than in the morning, but these differences were not significant (*p* = 0.08). Horn flies were the same before and after behavior observation (*p* = 0.55). Consequently, the effects of morning versus afternoon and start versus end were removed from the model.

Analyses of face fly numbers showed that very few face flies were observed throughout the study. During any given observation period, only one or two flies were counted on all animals in the group, resulting in a mean count of <1 face fly per cow (Figure 2D). Toward the end of the study, there was a slight increase in face fly counts, averaging one fly per cow. Fly numbers were significantly related to all weather variables (Table 1). Face fly counts also increased with temperature, though not as much as those of stable flies or horn flies. Added variable plots showed a slight decrease in face fly counts with an increasing humidity and an increase in counts with increasing wind speeds and precipitation. However, when adjusted for other factors, the relationships between weather components and face fly counts were weak. No significant differences were found in start versus end counts (*p* = 0.67), nor in morning versus afternoon counts (*p* = 0.08), so these effects were removed from the model. Cows harbored the same number of face flies regardless of parity (*p* = 0.69).

### 3.2. Variation in Behavior Frequencies

**Figure 5:**
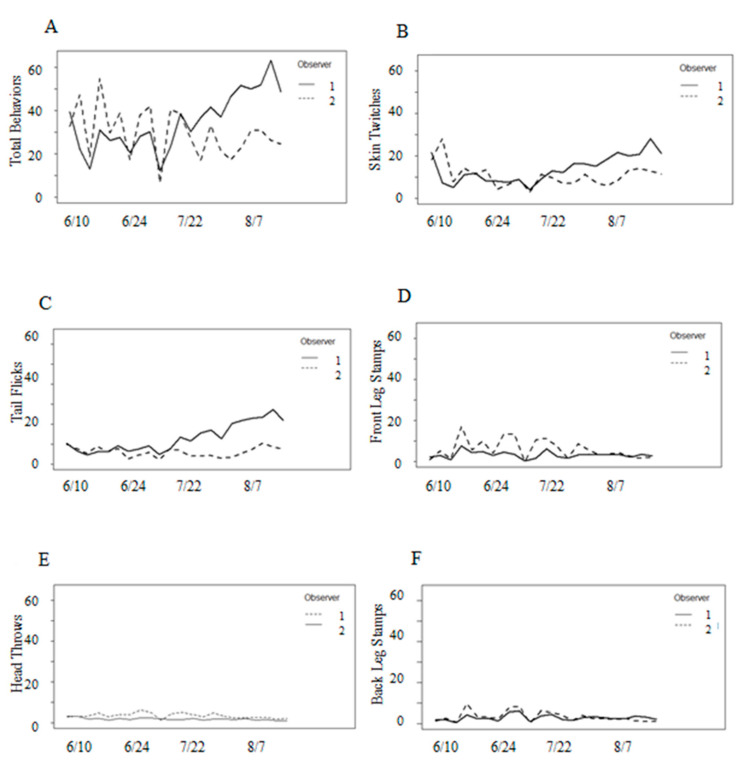
Mean behavior observations for observer 1 and 2: (**A**) all behaviors combined, (**B**) head throws, (**C**) skin twitches, (**D**) tail flicks, (**E**) front leg stamps, and (**F**) back leg stamps.

Because the random effects of groups and cows within groups accounted for less than 15% of the total variation, these effects were removed, and the analysis proceeded with fixed effects only. The frequencies of behaviors observed during the study were highly variable; some observation periods passed without observing any defensive behaviors, whereas observations were four to five times greater than the seasonal means in other periods (Table 2). Skin twitches were the most frequently observed defensive behavior, followed by tail flicks, front leg stamps, back leg stamps, and head throws (Table 2). A significant interaction between observer and date indicated that the observers counted different numbers of behaviors as the study progressed (Figure 5). 

**Figure 6:**
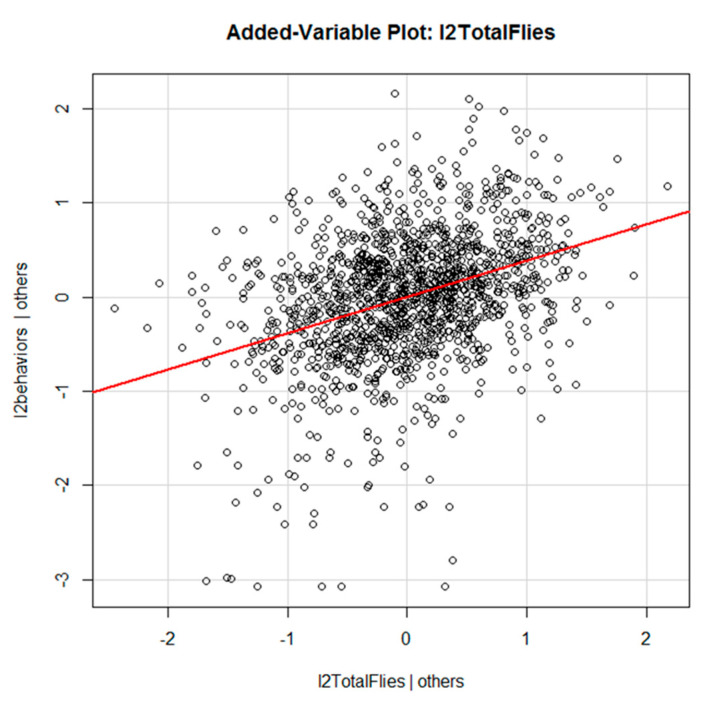
Added variable plot showing increase in behavior frequencies with increasing fly numbers, adjusting for other factors within the model. The red line is the regression line.

There was a very strong positive correlation between frequencies of defensive behaviors and adjusted fly counts, in that behaviors increased with fly count (Figure 6). Of temperature, humidity, wind, and precipitation, only temperature was significant to the total behavioral observations (Table 3). Stable flies and horn flies were both highly associated with total behavioral responses (Table 3), while face flies were not (*p* = 0.53). Behavior frequencies were independent of parity (*p* = 0.45), and so parity was removed from the model. This model was tested using a polynomial regression and we found no significant evidence of curvature or saturation for any fly species (*p* > 0.05). In the observed range of fly counts, the frequencies of defensive behaviors increased with flies, without any obvious curvature. An examination of the plots showed no clear pattern between the number of defensive behaviors and time period. Front and back leg stamps and head throws were consistent throughout the study, while skin twitches and tail flicks appeared to increase as the study progressed (Figure 5).

Temperature, humidity, date, observer, and horn flies and stable flies on both the front and back legs were associated with variation in all defensive behaviors when examined individually (Table 4). Skin twitches were most strongly related with horn flies and front leg stamps were most strongly related with stable flies (Table 4). Horn flies and stable flies were similarly related to tail flicks, back leg stamps, and head throws (Table 4). 

**Figure 7:**
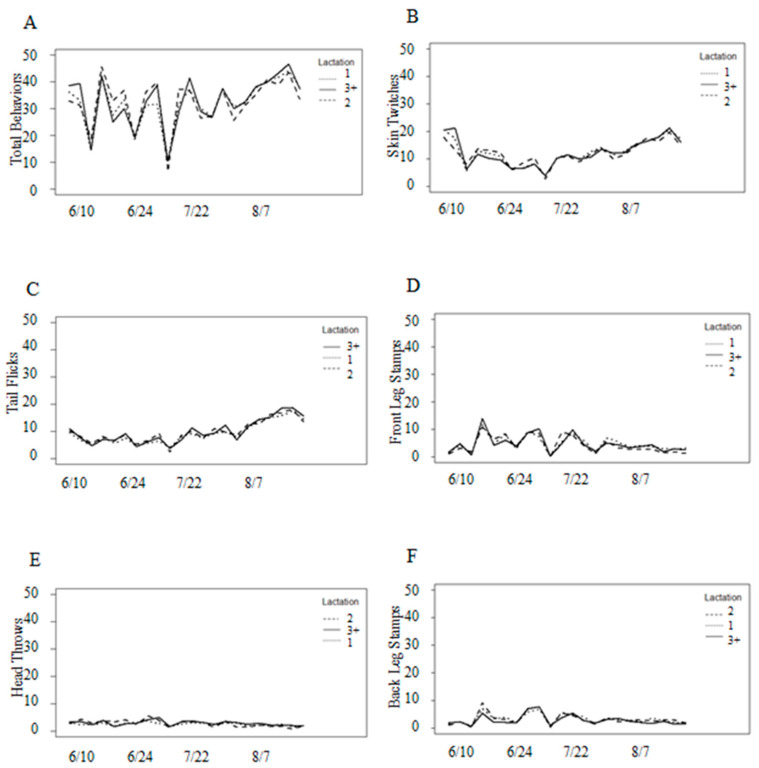
Behavior frequencies by parity (1, 2, or 3+). Parity was associated with (**A**) total behaviors, (**C**) front leg stamps, (**D**) tail flicks, and (**F**) head throws, but not with (**B**) skin twitches, nor (**E**) back leg stamps. There was no interaction between date and parity.

However, an examination of added variable plots indicated that the associations between weather variables and defensive behaviors were weak. Face flies were associated with front leg stamps, but independent of all other behaviors (Table 4). Parity was associated with front leg stamps, tail flicks, and head throws, but independent of skin twitches and back leg stamps (Table 4). There was no pattern throughout the study with behavior frequencies and parity despite younger cows hosting fewer horn flies and stable flies (Figure 7).

### 3.3. Milk Production

**Figure 8:**
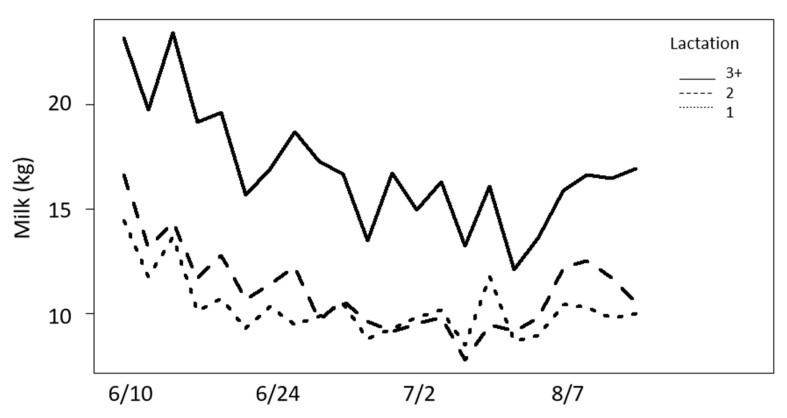
Daily milk production (kg) by cows in 1st lactation, 2nd lactation, and 3+ lactations in summer.

Milk production steadily decreased as the summer progressed and leveled off as the study concluded in August (Figure 8). Cows within groups as a random effect accounted for over 60% of the overall variation and so cows were retained in the statistical model. There were strong associations between milk production, parity, and days in milk. Older cows produced significantly more milk than cows in their first or second lactation. For each day in milk, we observed a decrease of 0.03 ± 0.01 kg of milk produced per day. The regression coefficients for production ranged from 0.10 to 0.13 for fly numbers on cows.

In the observed range of fly numbers, milk production was independent of the fly numbers of all three fly species combined (*p* = 0.16). Analyses were repeated for individual species. Horn flies were initially significant to milk production, but milk production was independent of behaviors (Table 5). However, when insignificant factors were removed to simplify the model, horn flies were no longer significant (*p* = 0.18), nor was the interaction between horn flies and date (*p* = 0.172). According to our minimally sufficient model, milk production was independent of any fly species and defensive behaviors. No decrease in milk production was observed with increasing behaviors, but when accounting for other factors, primarily lactation and DIM, this relationship was very weak.

## 4. Discussion

Varying numbers of all three species of flies were observed, with horn flies being the most frequently observed and face flies being the least frequently observed. There was a strong association between fly counts and defensive behaviors, with behaviors increasing with count. There was a weak association between fly counts, defensive behaviors, and weather variables, most notably an increase in counts and behaviors with an increasing temperature. In the observed range of fly counts, no associations were observed between milk production and fly counts or defensive behaviors. Previous research has focused on cattle behavioral responses to a single fly species. This study is the first to count three species of flies on free-roaming pastured cows to detect associations with defensive behaviors and production.

In northern regions, muscid flies are most active from May through to October, with peak activity mid-summer, depending on weather conditions. This study was conducted in the middle of the fly season, and we did not observe population fluctuations indicative of seasonal changes. Mullens et al. [15] tested for and found evidence of habituation, in that intensive behaviors (head throws and leg stamps) decreased as the season progressed. These intensive behaviors are immediate responses to stable fly feeding activity [3,16]. In contrast to Mullens et al. [15], intensive behaviors were consistent throughout the present study. The study was conducted in the middle of the fly season, and so the cows may have already habituated to fly presence by the beginning of the study. Flies were first observed on the cows approximately two months before the study’s start. During that time, the cows could have already adjusted to hosting flies before the study began. Although the counts occasionally reached over 150 flies on one animal, these numbers were not repeatedly observed. This lack of long-term exposure to high numbers of flies could be an explanation for not detecting any association between fly counts and milk production. 

Todd [14] noted that, under typical summer conditions, stable flies were most active between 11:00 am and 3:00 pm, and found that weather, especially temperature, was an important predictor of fly count. Similar to the current study, temperature was a consistently significant weather variable for both fly counts and behavior frequencies. The temperatures in this study ranged from 11 °C to 29 °C, well within the active range of muscid flies. During observation periods when the temperature fell below ~13 °C, few flies were counted, and the cows exhibited little to no defensive behaviors. Decreases in horn fly and face fly counts were observed with an increasing wind speed, which is consistent with previous studies. There was a strong relationship between defensive behaviors and weather conditions, notably increasing behavior with temperature. There was a slight increase in fly count as temperature increased, so observing more defensive behaviors during those times is expected. Another possible explanation is the effect of warmer weather on cow behavior. Cows are prone to heat stress, and when exposed to warmer weather conditions, were possibly more irritable and sensitive to fly activity. Decreases in fly numbers and defensive behaviors were observed during rainy periods. It is possible that horn flies, although present and counted, were not biting during rain. Cows may also be somewhat desensitized to fly activity due to rain.

The economic injury levels of stable flies on beef cattle are highly variable, with daily counts ranging from 25 [27] to 50 flies per cow [28]. Todd [14] found an index of irritability for stable fly numbers of up to 15 flies per animal. Fly numbers exceeding 15 did not result in an increase in irritation shown by behaviors. Unrest in cattle can be caused by feeding activity from even a few stable flies, as two to five flies per leg have been shown to cause reduced weight gain and feed efficiency [29]. According to Taylor et al. [26], when stable fly numbers range from 0 to 15 flies per leg, each additional fly causes daily milk losses of 0.22 kg per day. Furthermore, there may be blood loss results from hematophagy due to flies on cows. Blood loss may be a result of the defensive behaviors of cows. Only 15 flies per cow resulted in a loss of 15 mL of blood from the cows [30]. The cows in this study were obviously irritated by fly activity, but we were unable to detect such effects on milk production in the present study. 

Economic thresholds and injury levels are useful for producers to determine when intervention is needed, though these levels vary with fly species. Schwinghammer et al. [23] found that beef steers exposed to 100 to 500 horn flies showed increased physiological stress indicators, such as am increased heart rate, respiration, and rectal temperature. Irritation from horn flies can lead to decreased feed efficiency, weight gain, and milk production [31,32]. Treatment is generally recommended when populations exceed 200 flies per head, or 100 flies per side [33]. The average horn fly count in the current study was approximately 35 flies per side, ranging from less than 10 flies to 150 flies, well below this estimate. 

Virtually no face flies were observed in this study, with average counts of less than 1 fly per animal during the summer and counts never exceeding 10 flies per animal. There is little evidence that face fly infestations have a significant impact on milk production or quality [34,35]. Arends et al. [36] found no evidence of reduced feed efficiency or average daily gain on heifers infested with 13 or more face flies, and Schmidtmann et al. [34] did not detect effects on milk yield due to face fly numbers. Therefore, it is not surprising to observe little effect on production from face flies in the present study. 

Despite bearing similar fly loads, the cows reacted differently based on their parity. However, there was no consistently distinct pattern as to how younger or older cows reacted to varying levels of fly activity. In contrast, Mullens [15] observed fewer flies on younger cows, as well as more leg stamps when compared to older cows. In the observed range of fly counts, we found no clear evidence that younger cows were more sensitive to fly activity than older cows. Quite possibly, the younger cows exhibited more defensive behaviors, and therefore had fewer flies.

Some defensive behaviors may serve as a deterrent, such as skin twitches and tail flicks, while others, such as leg stamps and head throws, are more of a direct response to pain [3]. Such behaviors are seldom observed when nuisance flies are absent. The intensity of attacks varies with time of day and weather conditions. Flies are particularly active when winds are low and temperatures are high [14]. Hafez and Gamal-Eddin [37] reported that stable flies fed on the sunny side of the host at temperatures at or below 30 °C. At temperatures exceeding 30 °C, flies fed on the shaded side of the host or sought other sheltered locations as a form of thermoregulation. Temperatures did not exceed 30 °C in our study and the side of observation was random.

Skin twitches and tail flicks were observed more frequently than head throws or leg stamps. Dougherty et al. [16] also found that skin twitch responses were saturated at very low populations of stable flies. There was a strong relationship between fly counts and all the observed defensive behaviors. During observation periods with very few flies, the cows exhibited few to no defensive behaviors. Such observation periods typically occurred in the morning, with temperatures around 13 °C. 

Daily milk yields were independent of the fly numbers on the same cows the day before during this study. Significant factors impacting milk yield were days in milk and the cow’s parity. In our observed range of counts, fly load did not significantly impact milk yield, despite obvious irritation exhibited by the cows. Kientiz et al. [7] reported that the milk production of cows was similar for cows that moved through a fly trap compared to cows that were not moved through a fly trap, and milk production differences were not evident with low fly populations. Benefits in improved milk production may not be likely with low fly populations on grazing dairy cattle. Quite possibly, there were no effects of fly pressure on milk production in the current study because of the low fly populations observed in this grazing herd. Two hundred horn flies per cow is the accepted economic threshold level for beef cattle [33]. Jonsson and Mayer [38] reported that 30 horn flies or lower on dairy cattle would have no detrimental effect on milk production. The current study observed around 30 horn flies per cow, which may be a reason why there was no effect on milk production. However, in terms of behaviors, dairy cattle exhibit fly defensive behaviors at less than 100 horn flies per cow [39].

There was a strong association between fly counts and defensive behaviors, with higher frequencies of defensive behaviors with increasing numbers of flies. During some observation periods where very few flies were observed, no defensive behaviors were observed. Of weather variables, only temperature was associated with defensive behaviors when all combined. Other weather variables were statistically significant when analyzing individual defensive behaviors, though these relationships were weak. There was no consistent pattern throughout the study with behavior frequencies and parity, despite younger cows hosting fewer horn flies and stable flies on average. Quite possibly, the climate and sample size of cows might have impacted the results. Furthermore, the surrounding landscape (lands and ponds) or location relative to other livestock may have contributed to the number of flies observed on the cows.

## 5. Conclusions

The results of the current study indicate that, despite irritation, dairy cows can tolerate light to moderate fly loads without negative effects on milk production. However, further research is needed to investigate how the presence of additional species of nuisance flies can affect the current economic thresholds currently determined for a single species. Cows may not be able to tolerate higher fly loads when multiple species are present. In addition, further research is needed to better understand the impact of infestations from multiple species on cow comfort and productivity, especially in grazing settings. 

## Figures and Tables

**Table 1:** Results from ANOVA examining variation in log transformed counts of three species of muscid flies in summer 2014.

	F-Value	Degrees of Freedom	Coefficient	Standard Error
Stable Flies				
Temperature (°C)	3035.0	1, 42	0.15	0.004
Precipitation (mm)	71.1	1, 42	2.92	0.60
Humidity (%RH)	64.6	1, 42	−0.75	0.16
Location (front legs)	984.5	1, 42	0.75	0.02
Observer (1)	177.7	1, 42	0.44	0.12
Date	57.5	21, 42	--	--
Parity (2)	9.8	2, 42	0.23	0.14
Parity (3+)		2, 42	0.02	0.12
Observer × Date	23.4	21, 42	--	--
Parity × Date	1.9	42, 42	--	--
Horn Flies				
Temperature	81.2	1, 42	0.04	0.01
Precipitation	106.0	1, 42	10.20	1.84
Humidity	21.7	1, 42	−2.83	0.51
Wind	83.7	1, 42	1.00	0.18
Observer (2)	182.3	1, 42	−0.08	0.13
Date	30.5	21, 42	--	--
Parity (2)	38.7	2, 42	0.70	0.16
Parity (3+)			0.43	0.15
Observer × Date	34.0	21, 42	--	--
Parity × Date	2.2	42, 42	--	--
Face Flies				
Temperature	128.6	1, 42	0.02	0.00
Precipitation	13.2	1, 42	−1.90	0.99
Humidity	16.0	1, 42	0.56	0.28
Wind	46.1	1, 42	−0.21	0.09
Observer (2)	18.1	1, 42	0.09	0.1
Date	22.2	21, 42	--	--
Observer × Date	10.6	21, 42	--	--

**Table 2:** Summary statistics of tallied defensive behaviors.

Behavior	Mean	Median	Maximum	SD
Skin Twitch	16	10.5	84	9.0
Tail Flick	9.3	7.0	46	7.2
Front Leg Stamp	4.7	3.0	50	5.9
Head Throw	4.0	2.8	17	2.6
Back Leg Stamp	2.0	2.0	30	3.6

**Table 3:** Results from ANOVA examining variation in log transformed total behavior frequencies.

	F-Value	Degrees of Freedom	*p*-Value
Temperature	1205.2	1, 42	<0.001
Date	18.2	21, 42	<0.001
Observer	91.0	1, 42	<0.001
Stable Flies (front legs)	308.8	1, 42	<0.001
Stable Flies (back legs)	34.0	1, 42	<0.001
Horn Flies	39.8	1, 42	<0.001
Observer: Date	9.1	21, 42	<0.001
Date: SF (front legs)	7.1	21, 42	<0.001

**Table 4:** Results from ANOVA examining variation in log transformed individual defensive behaviors.

	F-Value	Degrees of Freedom	*p*-Value	Coefficient	SE
Skin Twitches					
Temperature	730.7	1, 42	<0.001	0.09	0.01
Humidity	15.1	1, 42	0.000	−0.83	0.92
Precipitation	62.8	1, 42	<0.001	2.74	3.2
Wind	49.7	1, 42	<0.001	0.35	0.31
Date	18.6	21, 42	<0.001	--	--
Observer 2	120.0	1, 42	<0.001	0.24	0.16
Stable flies (front legs)	126.1	1, 42	<0.001	0.03	0.08
Stable flies (back legs)	10.12	1, 42	0.003	0.05	0.03
Horn Flies	18.4	1, 42	0.000	0.09	0.03
Front Leg Stamp					
Temperature	897.2	1, 42	<0.001	0.14	0.01
Humidity	5.6	1, 42	0.023	−0.002	0.03
Wind	84.1	1, 42	<0.001	−0.16	0.12
Date	20.9	21, 42	<0.001	--	--
Observer 2	83.3	1, 42	<0.001	0.43	0.22
Parity (2) Parity (3+)	2.6 3.6	2, 42 2, 42	0.037 0.037	−0.20 −0.09	0.06 0.05
Stable flies (front legs)	224.0	1, 42	<0.001	0.15	0.11
Stable flies (back legs)	8.1	1, 42	0.007	0.02	0.03
Horn Flies	20.9	1, 42	<0.001	0.12	0.04
Face Flies	5.3	1, 42	0.026	0.07	0.04
Tail Flicks					
Temperature	447.0	1, 42	<0.001	0.07	0.01
Humidity	34.7	1, 42	<0.001	−0.01	0.93
Precipitation	54.9	1, 42	<0.001	−0.06	0.31
Wind	33.9	1, 42	<0.001	0.07	3.33
Date	25.9	21, 42	<0.001	--	--
Observer 2	670.9	1, 42	<0.001	0.08	0.16
Parity (2) Parity (3+)	4.1 4.1	2, 42 2, 42	0.025 0.0245	0.08 0.08	0.05 0.04
Stable flies (front legs)	114.2	1, 42	<0.001	0.13	0.08
Stable flies (back legs)	22.6	1, 42	<0.001	−0.01	0.03
Horn Flies	33.8	1, 42	<0.001	0.12	0.03
Back Leg Stamps					
Temperature	557.2	1, 42	<0.001	0.11	0.01
Humidity	5.7	1, 42	0.022	−0.02	0.03
Date	17.9	21, 42	<0.001	--	--
Observer 2	0.1	1, 42	0.807	0.09	0.21
Stable flies (front legs)	89.9	1, 42	<0.001	0.13	0.11
Stable flies (back legs)	19.9	1, 42	<0.001	0.11	0.34
Horn Flies	7.9	1, 42	0.007	0.1	0.34
Head Throw					
Temperature	117.4	1, 42	<0.001	0.04	0.01
Humidity	10.2	1, 42	0.003	−0.04	0.04
Wind	28.6	1, 42	<0.001	0.05	0.11
Date	8.1	21, 42	<0.001	--	--
Observer 2	165.7	1, 42	<0.001	0.23	0.21
Parity (2) Parity (3+)	7.8 7.8	2, 42 2, 42	0.001 0.001	0.07 0.15	0.06 0.05
Stable flies (front legs)	118.0	1, 42	<0.001	0.1	0.11
Stable flies (back legs)	5.1	1, 42	0.03	−0.15	0.11
Horn Flies	12.4	1, 42	0.001	0.13	0.03

**Table 5:** Results of ANOVA examining associations with milk production.

	F-Value	Degrees of Freedom	*p*-Value
Date	31.2	21, 1234	<0.001
Parity	32.1	2, 1234	<0.001
DIM	9.8	1, 1234	0.002
Stable flies	1.0	1, 1234	0.313
Horn flies	3.9	1, 1234	0.050
Face flies	0.0	1, 1234	0.922
Behaviors	0.0	1, 1234	0.930
Date: Behaviors	0.4	21, 1234	0.995
Date: Stable flies	1.4	21, 1234	0.111
Date: Horn flies	2.3	21, 1234	0.001

## Data Availability

Data available upon request from the corresponding author.
